# Depression and alcohol use disorder at antiretroviral therapy initiation led to disengagement from care in South Africa

**DOI:** 10.1371/journal.pone.0189820

**Published:** 2017-12-27

**Authors:** Cody Cichowitz, Noriah Maraba, Robin Hamilton, Salome Charalambous, Christopher J. Hoffmann

**Affiliations:** 1 Johns Hopkins University School of Medicine, Baltimore, Maryland, United States of America; 2 Aurum Institute, Johannesburg, South Africa; 3 Private practice clinical psychologist, Johannesburg, South Africa; 4 University of the Witwatersrand School of Public Health, Johannesburg, South Africa; 5 Division of Infectious Disease, Johns Hopkins University School of Medicine, Baltimore, Maryland, United States of America; Stellenbosch University, SOUTH AFRICA

## Abstract

We sought to assess mental health at the time of antiretroviral therapy (ART) initiation and subsequent retention in care over a six-month follow-up period. A total of 136 people living with HIV in South Africa were administered surveys measuring demographic information and mental health indicators at the time of ART initiation. Follow-up was completed via chart abstraction to assess for six-month outcomes of retention in care and viral suppression. At enrollment, 45/136 (33%), 67/136 (49%), and 45/136 (33%) participants screened positive for depression, anxiety, and alcohol use disorder, respectively. After six months of follow-up, 96/136 (71%) participants remained in care; 35/87 (40.2%) participants who remained in care had a level <50 copies/mL. Those with depression (49% vs. 77% retained; p < 0.01) and those with alcohol use disorder (52% vs. 76% retained; p < 0.01) were less likely to be retained in care. In multivariable logistic regression, depression OR 3.46 (95% CI: 1.33, 7.97; p < 0.01) and alcohol abuse OR 3.89 (95% CI: 1.70, 8.97; p < 0.01) were independently associated with loss from care. These results emphasize the importance of mental health on early ART outcomes and the HIV care continuum.

## Introduction

Worldwide there are approximately 36.7 million people living with HIV (PLHIV) of which 17 million have initiated antiretroviral therapy (ART) [1]. Health and prevention benefits from these programs depend on long-term engagement in care and adherence to ART. In sub-Saharan Africa (SSA) up to 24% of PLHIV drop out of care within one year of initiating treatment [2]. Improving retention in care is critical to the success of ART programs [3], and understanding and mitigating factors related to attrition from HIV care is an important area of study.

Throughout SSA, there is a high burden of mental illness amongst PLHIV [4–7], and mental illness can have a significant impact on the HIV-care continuum [6,8–10]. Some studies have estimated that up to half of PLHIV experience symptoms of mental illness [11]. The most common reported disorders include depression, anxiety, post-traumatic stress disorder, and alcohol use disorder [11]. Estimates place the prevalence of depressive symptoms, depression, and alcohol use at 31.2%, 18%, and between 8–42%, respectively [5,9]; differences in the reported prevalence often reflect the populations sampled and the assessment approaches used [12]. Studies from South Africa have replicated these findings [13–17].

It is well established that both depression and alcohol use negatively impact ART adherence [4,5,9,10,18]. Despite a growing body of literature on the prevalence of mental health and alcohol use disorders among PLHIV, gaps exist in understanding their impact on the HIV-care continuum in SSA [4,8]. Given the global push toward universal access to ART and the United Nations “90-90-90 goals” [19], continued research is needed to characterize the impact of mental health along the care continuum in order to inform ongoing efforts to promote retention in care.

The purpose of this prospective cohort study was twofold: (1) to describe the burden of mental illness and psychological distress present at the time of ART initiation, and (2) to assess the subsequent impact on retention in HIV care and viral suppression six months after ART initiation.

## Materials and methods

### Participants and setting

This study was a prospective, cohort study that recruited participants from February to June of 2013. Participants were recruited from a government-run primary care clinic in a densely populated peri-urban community in South Africa, close to Johannesburg. Sequential patients initiating ART during the study period were approached for recruitment by the research assistant. Participants were eligible for the study if they were 18 years of age or older, were attending an ART initiation visit (or pre-initiation ART counseling), were able to communicate in English, isiZulu, isiXhosa, seSotho, or Sepedi, and were able to provide written or witnessed oral consent. Participants were compensated for their time with a voucher worth approximately US$3, keeping with local remuneration rates.

The clinic from which participants were recruited provided standard HIV care as per the South African national guidelines. During the time frame of study recruitment, the ART initiation threshold in South Africa was a CD4 count <350 cells/mm^3^. The clinic utilized a tracing team to perform home visits for patients who missed scheduled visits. Tracers were sent to follow-up patients within 30 days of a missed visit and performed multiple home visits (if needed) in attempt to locate the patient and encourage the patient to return to care. Additionally, each person attending the clinic for ART initiation was given a unique medical record number and paper file during registration for ART or pre-ART care. This file was stored in the medical record department.

### Study procedures

After obtaining informed consent and completing study enrollment, a research assistant collected demographic and clinical information, conducted medical record reviews, and administered study surveys, including screening instruments for mental illness and alcohol use disorder. The research assistant had a degree in clinical psychology and was trained on the specific screening instruments by a practicing clinical psychologist. Abstracted data included age, sex, citizenship, primary language, employment status, and time spent living in the area near to the clinic. CD4 count results were recorded from the medical records at the time of enrollment. Participants’ paper medical files were reviewed between seven and nine months after study enrollment to assess six-month outcomes. If the file was not located, up to five attempts on different days were made to locate the file.

### Survey instruments

We selected instruments that were previously used in South Africa or SSA and validated in these settings. The Hospital Anxiety and Depression Scale (HADS) was used to screen for anxiety and depression. This scale has 14 total questions, seven assessing for symptoms of depression and seven assessing for symptoms of anxiety. A cut-off score of eight was used to indicate a positive screening result for either construct, based on prior publications [17,20–22]. The CAGE questionnaire, a four question-screening instrument, was used to screen for alcohol use disorder. Questions were modified from their original form and asked in present tense to screen for active alcohol use (i.e. have you felt the need to cut down on your drinking? Do you feel annoyed by people complaining about your drinking?). A cutoff score of two or more was used to define a positive screening result [23–27]. The AIDS-related Stigma Scale, a nine-question scale, was used to screen for internalized stigma and analyzed as a continuous variable ranging from zero to nine. A higher score represents higher levels of stigma [28–30]. Finally, the HIV/AIDS-Targeted Quality of Life (HAT-QoL) instrument was used to quantify the impact of HIV on participant’s quality of life [31–34]. The HAT-QoL has 42 total questions that assess quality of life across nine domains. The score on each domain of the HAT-QoL and the aggregate score were presented as a scaled sum out of 100, consistent with prior studies. A specific domain of the HAT-QoL, *Disclosure Worries*, was used to evaluate participants’ concerns about disclosing their HIV status. Participants with psychological screening tests above the pre-specified cut-offs were referred to a social worker or clinical psychologist employed by the local health system for further assessment and counseling.

### Primary outcomes

The primary outcome data included: adherence to scheduled clinic appointments, medication refills, and HIV RNA (viral load) data. Participants were defined as disengaged from care if they met one of two criteria: (1) had not attended an appointment or received medication for three or more months, or (2) the file was either not located by the study team or was noted to have been removed by the clinic staff because the patient had not been to the clinic in the past six months. The definition of care disengagement was chosen in order to be consistent with prior studies measuring retention in low- and middle-income countries [2] and the operational definition of treatment default used by the South African Department of Health [35]. In addition to tracing clients who missed appointments, the clinic noted transfers to other clinics and patient deaths in the paper patient files and/or a clinic register. Viral suppression was defined as an HIV RNA less than 50 copies/mL.

### Statistical analysis

An *a priori* sample size goal of 200 was derived to detect a 10% or greater difference in outcomes in those with mental health disorders. For a variety of operational issues this goal was not achieved; issues included a period of time when the study clinic stopped new ART initiation due to staff shortages, a period of time during which phlebotomy supplies were unavailable (necessary for CD4-based ART initiation), and a period of time when the local supplies of ART medications were limited and fewer people were initiated. Descriptive statistics, including proportions for categorical variables and median values and inter-quartile ranges for continuous variables, were used to describe the study population and the results of surveys. Spearman correlation coefficients were calculated between the survey instruments to assess for the presence of simultaneously occurring disorders and co-linearity between variables. Bivariate analysis was completed to look for associations between demographic, laboratory, and survey data and retention in care six months after enrollment using Chi-squared and Mann-Whitney U tests. Age and any variables with p-values ≤ 0.05 in bivariate analysis were included in a multivariable logistic regression. All analyses were completed in STATA 13 (Stata Corporation, College Station, Texas).

### Ethics statement

This research was conducted according to the principles expressed in the Declaration of Helsinki; written or witnessed oral informed consent was obtained from all participants prior to study procedures. The study was approved by the institutional review boards of the Johns Hopkins University School of Medicine and the University of the Witwatersrand.

## Results

166 patients visited the clinic to initiate ART during the study period; 136 participants enrolled, 28 declined due to time constraints or unspecified reasons, and two were too ill to participate. Of the 136 participants, 67 (49%) were female and the median age was 37 (IQR: 31, 43) (Table 1). The median CD4 count prior to ART initiation was 236 (IQR: 101, 308). Most participants were South African citizens (86%) and had lived in the area around the clinic for more than two years (84.6%).

**Table 1:** Participant characteristics at time of ART initiation.

Variable	#/n	%
Gender:		
Female	67/136	49.3%
Age:		
Median (IQR)	37 (30.5, 43)	
SA Citizen:		
Yes	117/136	86.0%
Lived in area for > 2 years:		
Yes	115/136	84.6%
Employed or self-employed:		
Yes	73/136	53.7%
CD4 Count (cells/mm^3^)^a^:		
Median (IQR)	236 (101, 308)	
0–100	24/96	25.0%
100–200	14/96	14.6%
>200	58/96	60.4%

^a^Limited laboratory data were available at time of enrollment; 96/136 participants had CD4 counts recorded.

At enrollment, 45 (33%) of participants had a score ≥ 8 on the HADS Depression Scale, 67 (49%) had a score ≥ 8 of the HADS Anxiety Scale, and 45 (33%) participants screened positive for alcohol use disorder with a CAGE score ≥ 2 (Table 2). On the AIDS-related Stigma Scale, 34 (25%) endorsed two or more elements of stigma. On the HAT-QoL, the median score was 64.5 out of 100, with the most distress present on the domains of *Overall Function*, *Sexual Function*, *Disclosure Worries*, and *Financial Worries*.

**Table 2:** Mental health indicators at ART initiation (n = 136).

Survey	Min	Max	Median (IQR)	Proportion above cutoff #/n (%):
HADS Depression^a^ (out of 21)	0	21	5 (2.5; 8)	45/136 (33%)
HADS Anxiety^a^ (out of 21)	0	21	7 (3.5; 11.5)	67/136 (49%)
CAGE^b^ (out of 4)	0	4	0 (0; 2)	45/136 (33%)
Stigma^c^ (out of 9)	0	7	1 (0; 2)	
HAT-QOL Total^d^ (out of 100)	29.7	97.2	64.5 (56.6, 71.0)	
Overall Function (out of 100)	20.0	100.0	63.3 (53.3, 76.7)	
Sexual Function (out of 100)	20.0	100.0	60 (20, 100)	
Disclosure Worries (out of 100)	20.0	100.0	60 (48, 80)	
Health Worries (out of 100)	20.0	100.0	65 (47.5, 75)	
Financial Worries (out of 100)	20.0	100.0	46.7 (26.7, 73.3)	
HIV Mastery (out of 100)	20.0	100.0	70 (40, 90)	
Life Satisfaction (out of 100)	20.0	100.0	65 (55, 80)	
Provider Trust (out of 100)	20.0	100.0	80 (73.3, 100)	

^a^HADS: higher scores imply more symptoms and a cutoff of ≥8 used in screening for depression and anxiety.

^b^CAGE: A cutoff score of ≥2 was used to screen for alcohol use disorder.

^c^AIDS-related Stigma Scale: higher scores imply more experienced stigma.

^d^HAT-QoL: lower scores imply a lower quality of life. The composite score and each domain is presented as a scaled score out of 100.

Several of the instruments screening for disorders showed correlations with each other (Table 3). Depression was moderately correlated with anxiety (correlation coefficient 0.50) and quality of life (correlation coefficient -0.57). Anxiety was also moderately correlated with quality of life (correlation coefficient -0.47). Internalized stigma was mildly correlated with depression, anxiety, disclosure worries, and quality of life (correlation coefficients of 0.30, 0.29, -0.40, and -0.40 respectively). Alcohol use disorder was not associated with any other instrument scores.

**Table 3:** Correlations^a^ between mental health indicators.

	HADS Depression^b^	HADS Anxiety^b^	CAGE^b^	Stigma^b^	Disclosure Worries^c^	HAT-QoL Total Score^c^
HADS Depression^b^						
HADS Anxiety^b^	0.50^d^					
CAGE^b^	0.07	0.03				
Stigma^b^	0.30^d^	0.29^d^	0.06			
Disclosure Worries^c^	-0.14	-0.21^d^	0.01	-0.40^d^		
HAT-Qol Total Score^c^	-0.57^d^	-0.47^d^	0.05	-0.40^d^		

^a^Spearman correlation coefficients.

^b^Higher scores indicate more symptoms.

^c^Lower scores indicate more disclosure worries or lower quality of life.

^d^P-value ≤ 0.05.

At six-month follow up, 92/136 (67.6%) participants were retained in care and the remaining 44/136 (32.4%) participants met the definition of lost from care. No participant deaths or clinic transfers were documented in the patient files. HIV RNA results were available for 87/92 (94.6%) participants who were active in care at six months. Of the participants who remained in care and who had HIV RNA results, 35/87 (40.2%) had a level <50 copies/mL.

In bivariate analysis (Table 4), loss from care was associated with depression (49% retained when HADS score ≥ 8 vs. 77% retained when HADS score < 8, p < 0.01), alcohol use disorder (52% retained when CAGE score ≥ 2 vs. 76% retained when CAGE score < 2, p < 0.01), higher levels of internalized stigma (p = 0.01), and decreased quality of life (p = 0.03). The remaining screening instruments, including the HADS Anxiety Scale, were not associated with dropping out of care. In the multivariable logistic regression model (Table 5), depression, OR 3.46 (95% CI: 1.33, 7.97; p < 0.01), and alcohol use disorder, OR 3.89 (95% CI: 1.70, 8.97; p < 0.01), were independently associated with disengagement from care.

**Table 4:** Factors associated with retention in care.

**Variable**	**Outcome: Clinic Activity in Last 3 Months (n = 136)**		
	**n**	**Active n (%)**	**P-value**^a^
Gender:			
Male	69	44 (64)	.33
Female	67	48 (72)	
Citizenship:			
South African	117	81 (69)	.33
Foreign	19	11 (57)	
Duration of residence in area:			
<2 years	21	14 (67)	.92
≥2 years	115	78 (68)	
Employment status:			
Employed	73	49 (36)	.89
Unemployed	63	43 (32)	
CD4 count (cells/mm^3^)^c^:			
<100	24	22 (92)	.62
100–199	14	13 (93)	
≥ 200	58	56 (97)	
HADS Depression ^d^:			
< 8	91	70 (77)	< .01
≥ 8	45	22 (49)	
HADS Anxiety ^d^:			
< 8	69	46 (67)	.80
≥ 8	67	46 (69)	
CAGE ^d^:			
< 2	88	67 (76)	< .01
≥ 2	48	25 (52)	
**Variable**	**Inactive (n = 44): Median (IQR)**	**Active (n = 92): Median (IQR)**	**P-value**^b^
Age	38 (33, 41)	36 (30, 43)	.37
Stigma (out of 9)^d^	1 (0, 3)	1 (0, 1)	.01
Disclosure Worries (out of 100)^e^	60 (40, 76)	60 (48, 82)	.45
HAT-QoL Total (out of 100)^e^	62 (53, 67)	66 (57, 73)	.03

^a^ Chi-squared test between the outcome and the variables listed in each row.

^b^ Mann–Whitney U (rank-sum) test between the outcome and the variables listed in each row.

^c^ Limited laboratory data was available. 96/136 participants had CD4 counts recorded.

^d^Higher scores indicate more symptoms

^e^Lower scores indicate lower quality of life.

**Table 5:** Predictors of disengagement from care—multivariable logistic regression^a^ (n = 136).

Variable	OR (95% CI)	P-value
Age (per 5 year increase)	1.03 (0.97, 1.08)	.31
HADS Depression ≥ 8	3.46 (1.33, 7.97)	< .01
CAGE ≥ 2	3.89 (1.70, 8.97)	< .01
Stigma (per one point increase)	1.24 (0.92, 1.64)	.11

^a^Logistic regression evaluating predictors of **disengagement** from HIV care within the first six months after ART initiation. Age was included in this model as a control, along with any variable found to have a statically significant association (p ≤ .05) in bivariate analysis. HAT-QoL was excluded from the model due to a high correlation with both depression and stigma.

We did not identify any significant associations between HIV RNA results and instrument scores (S1 Table), potentially due to the small number of participants with laboratory data and due to loss from care among those with depression, alcohol use disorder, and higher stigma scores.

## Discussion

In this prospective cohort study, we recruited 136 PLHIV initiating ART and found a significant burden of psychological distress and alcohol use disorder. Higher scores on measures of depression and alcohol use were strongly associated with disengagement from care within the first six months after initiation ART.

Prior to ART initiation, a large proportion of participants screened positive for depression (33%), anxiety (49%), and alcohol use disorder (33%). Prior studies of PLHIV in South Africa have reported similarly high prevalence of these disorders with reported ranges of depression, anxiety, and alcohol use disorder being between 12–45%, 17–40%, and 18–35%, respectively [13–17]. Additionally, our results indicate that PLHIV face significant levels of internalized stigma and impaired quality of life at the time of ART initiation. This was particularly true with respect to overall functional status, sexual function, disclosure worries, and financial insecurity, findings consistent with prior studies [30,34].

We found that depression, anxiety, stigma, disclosure, and quality of life measures were modestly correlated. The overlap of these disorders speaks to the complexity of living with HIV. CAGE scores were not correlated with other measures suggesting that alcohol use affected both those with and without mental health disorders.

Overall, there was high lost to follow up after ART initiation (32.4%) and low rates of viral suppression (40.2%). A recent review and meta-analysis reported six-month retention to be 85% in South Africa with a range in six-month retention from 66 to 100% [2]. The proportion of participants retained in care is within the lower range of the published literature, but reasonable considering that participants were recruited from a public clinic in a peri-urban area with high population mobility. Moreover, it is possible that medication stock-outs, laboratory supply stock-outs, and labor unrest may have contributed to loss from care during the period of study and follow up.

Alcohol use disorder was strongly associated with disengagement from care with an OR of 3.89 (95% CI: 1.70, 8.97; p < 0.01). The effect of alcohol use on retention in care was independent from the similarly strong association between depression and loss from care, OR 3.46 (95% CI: 1.33, 7.97; p < 0.01). While alcohol use and depression have been associated with worse retention in care in the United States [36,37], prospectively replicating this association in South Africa is an important finding. It has been described that depression and alcohol impact adherence in SSA [5,9,10,18], yet these data show that alcohol use and depression have a significant impact on early retention in care.

In several disease states, including HIV, depression has been reported to reduce self-efficacy and self-care, two important constructs associated with retention in care and adherence to ART [38–40]. The literature regarding anxiety and self-efficacy is heterogeneous, with several studies from the heart failure field suggesting that anxiety is not associated with changes in self-efficacy [41] or may even be associated with increased self-efficacy after controlling for depression [42,43].

Within this study population, loss from care among participants with depression or alcohol use disorder was substantial, leading to an insufficient number of participants with either disorder to adequately assess for an association with ART adherence. While it is likely that adherence was also compromised among those with depression or alcohol disorder, as has been previously reported [5,10,18], these groups had been largely lost from care before adherence could be estimated from the measurement of HIV RNA, six months after ART initiation.

The strengths of this study are that it was a prospective, cohort study that assessed the relationship between measures of mental health on clinical outcomes six-months after ART initiation. The instruments used in this study were previously used in South Africa and found to have good validity and reliability. There are several important limitations. First, we used screening instruments rather than formal interviews to evaluate for mental illness. Second, limited laboratory data were available for participants (either as a result of the participant not attending a visit needed for laboratory testing or a failure in clinical services to perform the testing). Consequently, we were unable to fully evaluate the effects of mental health on viral suppression. Furthermore, it is likely that the subset of participants with HIV RNA data available represents a biased sample of the study population due to higher rates of disengagement from care in those with psychological distress. Third, it is possible that disengagement from care was overestimated due to the definition used (going greater than 90 days without returning to the clinic); some participants categorized as lost to follow up may have returned to care after the period of study follow up. Fourth and most importantly, we relied on medical record review for our prospective follow up. While the clinic had several mechanisms in place to trace patients who dropped out of care and to document deaths and transfers, there were likely gaps in documentation that led to a failure to identify some clinic-to-clinic transfers as well as most deaths. Finally, our sample size was small; however our goal was to assess whether mental illness may have a major potential role with large effect sizes. Our findings support this hypothesis.

## Conclusions

Identifying a high burden of mental illness and prospectively finding associations between retention in care and alcohol abuse and depression at the time of ART initiation is an important result. The presence of these disorders at the time of ART-initiation indicates their early impact on the HIV-care continuum. These psychological factors may contribute even more to attrition at even earlier stages in the HIV care continuum (before or after testing for HIV), suggesting a potential need to screen for and treat these disorders at or before the time of HIV testing [7,13]. Additional studies are needed to characterize cost-effective mental health interventions with the greatest impact on the care continuum.

## Supporting information

S1 Table Factors associated with viral suppression. (DOCX)

S1 Data De-identified version of the data collected and analyzed in this study. (CSV)

## References

[1] AIDS JUNPOH. Global AIDS update 2016. Geneva; 2016.

[2] Fox MP, Rosen S. Retention of Adult Patients on Antiretroviral Therapy in Low- and Middle-Income Countries: Systematic Review and Meta-analysis 2008–2013. J Acquir Immune Defic Syndr. 2015 5 1;69(1):98–108. 25942461 10.1097/QAI.0000000000000553 PMC4422218

[3] Shah M, Risher K, Berry SA, Dowdy DW. The Epidemiologic and Economic Impact of Improving HIV Testing, Linkage, and Retention in Care in the United States. Clin Infect Dis. 2015 12 24;62(2):220–9. doi: 10.1093/cid/civ801 26362321 10.1093/cid/civ801 PMC4690480

[4] Mayston R, Kinyanda E, Chishinga N, Prince M, Patel V. Mental disorder and the outcome of HIV/AIDS in low-income and middle-income countries. AIDS. 2012 12;26:S117–35. doi: 10.1097/QAD.0b013e32835bde0f 23303434 10.1097/QAD.0b013e32835bde0f

[5] Nakimuli-Mpungu E, Bass JK, Alexandre P, Mills EJ, Musisi S, Ram M, et al Depression, Alcohol Use and Adherence to Antiretroviral Therapy in Sub-Saharan Africa: A Systematic Review. AIDS Behav. 2011 11 25;16(8):2101–18. 10.1007/s10461-011-0087-8 22116638

[6] Nanni MG, Caruso R, Mitchell AJ, Meggiolaro E, Grassi L. Depression in HIV Infected Patients: a Review. Curr Psychiatry Rep. 2014 11 21;17(1):e4422006–12. 10.1007/s11920-014-0530-4 25413636

[7] Kagee A, Saal W, De Villiers L, Sefatsa M, Bantjes J. The Prevalence of Common Mental Disorders Among South Africans Seeking HIV Testing. AIDS Behav. Springer US; 2016 5 17;21(6):1511–7. 10.1007/s10461-016-1428-4 PMC5114161 27188430

[8] Abas M, Ali G-C, Nakimuli-Mpungu E, Chibanda D. Depression in people living with HIV in sub-Saharan Africa: time to act. Trop Med Int Health. 5 ed. 2014 10 16;19(12):1392–6. doi: 10.1111/tmi.12382 25319189 10.1111/tmi.12382

[9] Williams EC, Hahn JA, Saitz R, Bryant K, Lira MC, Samet JH. Alcohol Use and Human Immunodeficiency Virus (HIV) Infection: Current Knowledge, Implications, and Future Directions. Alcohol Clin Exp Res. 2016 9 22;40(10):2056–72. doi: 10.1111/acer.13204 27696523 10.1111/acer.13204 PMC5119641

[10] Vagenas P, Azar MM, Copenhaver MM, Springer SA, Molina PE, Altice FL. The Impact of Alcohol Use and Related Disorders on the HIV Continuum of Care: a Systematic Review. Curr HIV/AIDS Rep. 2015 9 28;12(4):421–36. doi: 10.1007/s11904-015-0285-5 26412084 10.1007/s11904-015-0285-5 PMC4643391

[11] Brandt R. The mental health of people living with HIV/AIDS in Africa: a systematic review. African Journal of AIDS Research. 2009 6;8(2):123–33. doi: 10.2989/AJAR.2009.8.2.1.853 25875564 10.2989/AJAR.2009.8.2.1.853

[12] Tsai AC. Reliability and Validity of Depression Assessment Among Persons With HIV in Sub-Saharan Africa. JAIDS Journal of Acquired Immune Deficiency Syndromes. 2014 8;66(5):503–11. doi: 10.1097/QAI.0000000000000210 24853307 10.1097/QAI.0000000000000210 PMC4096047

[13] Cholera R, Gaynes BN, Pence BW, Bassett J, Qangule N, Macphail C, et al Validity of the patient health questionnaire-9 to screen for depression in a high-HIV burden primary healthcare clinic in Johannesburg, South Africa. Journal of Affective Disorders. 2014 10;167:160–6. doi: 10.1016/j.jad.2014.06.003 24972364 10.1016/j.jad.2014.06.003 PMC4264106

[14] Breuer E, Stoloff K, Myer L, Seedat S, Stein DJ, Joska JA. The Validity of the Substance Abuse and Mental Illness Symptom Screener (SAMISS) in People Living with HIV/AIDS in Primary HIV Care in Cape Town, South Africa. AIDS Behav. Springer US; 2014 1 23;18(6):1133–41. 10.1007/s10461-014-0698-y 24452497

[15] Myer L, Smit J, Roux LL, Parker S, Stein DJ, Seedat S. Common Mental Disorders among HIV-Infected Individuals in South Africa: Prevalence, Predictors, and Validation of Brief Psychiatric Rating Scales. AIDS Patient Care and STDs. 2008 2;22(2):147–58. doi: 10.1089/apc.2007.0102 18260806 10.1089/apc.2007.0102

[16] Joska JA, Fincham DS, Stein DJ, Paul RH, Seedat S. Clinical Correlates of HIV-Associated Neurocognitive Disorders in South Africa. AIDS Behav. 2009 3 27;14(2):371–8. doi: 10.1007/s10461-009-9538-x 19326205 10.1007/s10461-009-9538-x

[17] Pappin M, Wouters E, Booysen FLR. Anxiety and depression amongst patients enrolled in a public sector antiretroviral treatment programme in South Africa: a cross-sectional study. Eleventhth. 2012;12(1):244. 10.1186/1471-2458-12-244 PMC3378441 22452846

[18] Gonzalez JS, Batchelder AW, Psaros C, Safren SA. Depression and HIV/AIDS Treatment Nonadherence: A Review and Meta-analysis. JAIDS Journal of Acquired Immune Deficiency Syndromes. 2011 10;58(2):181–7. doi: 10.1097/QAI.0b013e31822d490a 21857529 10.1097/QAI.0b013e31822d490a PMC3858003

[19] AIDS JUNPOH. 90-90-90: an ambitious treatment target to help end the AIDS epidemic. Geneva: UNAIDS; 2014.

[20] Wouters E, Booysen FLR, Ponnet K, Baron Van Loon F. Wording Effects and the Factor Structure of the Hospital Anxiety & Depression Scale in HIV/AIDS Patients on Antiretroviral Treatment in South Africa. Graham SM, editor. PLoS ONE. Public Library of Science; 2012 4 20;7(4):e34881–10. 10.1371/journal.pone.0034881 PMC3335020 22536338

[21] Wouters E, Masquillier C, Booysen FR. The Importance of the Family: A Longitudinal Study of the Predictors of Depression in HIV Patients in South Africa. AIDS Behav. Springer US; 2016 1 14;:1–13. 10.1007/s10461-016-1294-0 26781870

[22] Reda AA. Reliability and Validity of the Ethiopian Version of the Hospital Anxiety and Depression Scale (HADS) in HIV Infected Patients. Mitchell A, editor. PLoS ONE. 2011 1 25;6(1):e16049–6. doi: 10.1371/journal.pone.0016049 21283565 10.1371/journal.pone.0016049 PMC3026786

[23] Ewing JA. Detecting alcoholism. The CAGE questionnaire. JAMA. 1984 10 12;252(14):1905–7. 6471323 10.1001/jama.252.14.1905

[24] Dhalla S, Kopec JA. The CAGE Questionnaire for Alcohol Misuse: A Review of Reliability and Validity Studies. Clinical & Investigative Medicine. 2007 2 11;30(1):33–41. 17716538 10.25011/cim.v30i1.447

[25] Claassen JN. The benefits of the CAGE as a screening tool for alcoholism in a closed rural South African community. South African medical journal. 1999. 10554635

[26] Parry CDH, Plüddemann A, Steyn K, Bradshaw D, Norman R, Laubscher R. Alcohol use in South Africa: findings from the first Demographic and Health Survey (1998). J Stud Alcohol. 2005 1;66(1):91–7. 15830908 10.15288/jsa.2005.66.91

[27] Rendall-Mkosi K, Morojele N, London L, Moodley S, Singh C, Girdler-Brown B. A randomized controlled trial of motivational interviewing to prevent risk for an alcohol-exposed pregnancy in the Western Cape, South Africa. Addiction. 2013 4;108(4):725–32. doi: 10.1111/add.12081 23216868 10.1111/add.12081

[28] Kalichman SC, Simbayi LC, Jooste S, Toefy Y, Cain D, Cherry C, et al Development of a Brief Scale to Measure AIDS-Related Stigma in South Africa. AIDS Behav. Kluwer Academic Publishers-Plenum Publishers; 2005 6;9(2):135–43. 10.1007/s10461-005-3895-x 15933833

[29] Tsai AC, Weiser SD, Steward WT, Mukiibi NFB, Kawuma A, Kembabazi A, et al Evidence for the Reliability and Validity of the Internalized AIDS-Related Stigma Scale in Rural Uganda. AIDS Behav. 2012 8 7;17(1):427–33. 10.1007/s10461-012-0281-3 PMC3505811 22869104

[30] Simbayi LC, Kalichman S, Strebel A, Cloete A, Henda N, Mqeketo A. Internalized stigma, discrimination, and depression among men and women living with HIV/AIDS in Cape Town, South Africa. Social Science & Medicine. 2007 5;64(9):1823–31. 17337318 10.1016/j.socscimed.2007.01.006 PMC4271649

[31] Oparah AC, Soni JS, Arinze HI, Chiazor IE. Patient-Reported Quality of Life During Antiretroviral Therapy in a Nigerian Hospital. Value in Health Regional Issues. 2013 9;2(2):254–8. 10.1016/j.vhri.2013.07.004 29702873

[32] Holmes WC, Shea JA. A new HIV/AIDS-targeted quality of life (HAT-QoL) instrument: development, reliability, and validity. 1998;36(2):138–54. 10.1097/00005650-199802000-00004 9475469

[33] Taylor TN, Dolezal C, Tross S, Holmes WC. Reliability and validity of two HIV/AIDS-specific quality of life instruments adapted for use in HIV-positive Zimbabweans. AIDS Care. 2009 5 14;21(5):598–607. doi: 10.1080/09540120802302574 19444668 10.1080/09540120802302574 PMC5576544

[34] Soares GB, Garbin CAS, Rovida TAS, Garbin AJÍ. Quality of life of people living with HIV/AIDS treated by the specialized service in Vitória-ES, Brazil. Ciênc saúde coletiva. 2015;20(4):1075–84. 10.1590/1413-81232015204.00522014 25923619

[35] National Department of Health. National Consolidated Guidelines for the prevention of mother-to-child transmission of HIV (PMTCT) and the management of HIV in children, adolescents, and adults. South Africa National Department of Health. 2015 Apr; 1–136.

[36] Monroe AK, Lau B, Mugavero MJ, Mathews WC, Mayer KH, Napravnik S, et al Heavy Alcohol Use Is Associated With Worse Retention in HIV Care. J Acquir Immune Defic Syndr. 2016 12 1;73(4):419–25. doi: 10.1097/QAI.0000000000001083 27243904 10.1097/QAI.0000000000001083 PMC5085857

[37] Zuniga JA, Yoo-Jeong M, Dai T, Guo Y, Waldrop-Valverde D. The Role of Depression in Retention in Care for Persons Living with HIV. AIDS Patient Care and STDs. 2016 1;30(1):34–8. doi: 10.1089/apc.2015.0214 26544915 10.1089/apc.2015.0214 PMC4717497

[38] Tatum AK, Houston E. Examining the interplay between depression, motivation, and antiretroviral therapy adherence: a social cognitive approach. AIDS Care. Taylor & Francis; 2016 8 29;29(3):306–10. 10.1080/09540121.2016.1220481 27684790

[39] Gonzalez JS, Fisher L, Polonsky WH. Depression in Diabetes: Have We Been Missing Something Important? Diabetes Care. 2010 12 29;34(1):236–9. 10.2337/dc10-1970 PMC3005471 21193621

[40] Schinckus L, Dangoisse F, Van den Broucke S, Mikolajczak M. When knowing is not enough: Emotional distress and depression reduce the positive effects of health literacy on diabetes self-management. Patient Education and Counseling. Elsevier Ireland Ltd; 2017 8 27;:1–7. 10.1016/j.pec.2017.08.006 28855062

[41] Müller-Tasch T, Löwe B, Lossnitzer N, Frankenstein L, Täger T, Haass M, et al Anxiety and self-care behaviour in patients with chronic systolic heart failure: A multivariate model. Eur J Cardiovasc Nurs. 2017 7 1;5402:1474515117722255. 10.1177/1474515117722255 28718661

[42] Bauer LK, Caro MA, Beach SR, Mastromauro CA, Lenihan E, Januzzi JL, et al Effects of Depression and Anxiety Improvement on Adherence to Medication and Health Behaviors in Recently Hospitalized Cardiac Patients. Am J Cardiol. Elsevier Inc; 2012 5 1;109(9):1266–71. 10.1016/j.amjcard.2011.12.017 22325974

[43] Kessing D, Denollet J, Widdershoven J, Kupper N. Psychological Determinants of Heart Failure Self-Care. Psychosomatic Medicine. 2016 5;78(4):412–31. doi: 10.1097/PSY.0000000000000270 27082055 10.1097/PSY.0000000000000270

