# Supplementary material for: Depression and alcohol use disorder at antiretroviral therapy initiation led to disengagement from care in South Africa
Source: PLoS One. 2017 Dec 27;12(12):e0189820. doi: 10.1371/journal.pone.0189820 (PMC5744960; doi:10.1371/journal.pone.0189820)
Supplement: S1 Table — (DOCX) [file pone.0189820.s001.docx]

**S1 Table: Factors associated with viral suppression**

| **Variable** | **Outcome: Viral Suppression (n = 87)** | | |
| --- | --- | --- | --- |
|  | **n** | **VL≤ 50: n (%)** | **P-value^a^** |
| Gender: |  |  |  |
| Male | 43 | 19 (44) | .46 |
| Female | 44 | 16 (36) |  |
| Citizenship: |  |  |  |
| South African | 9 | 1 (11) | .06 |
| Foreign | 78 | 34 (44) |  |
| Duration of residence in area: |  |  |  |
| <2 years | 13 | 5 (39) | .89 |
| ≥2 years | 74 | 30 (41) |  |
| Employment status: |  |  |  |
| Employed | 45 | 20 (44) | .41 |
| Unemployed |  |  |  |
| CD4 count (cells/mm^3^): |  |  |  |
| <100 | 18 | 3 (17) | .07 |
| 100-199 | 12 | 5 (42) |  |
| ≥ 200 | 57 | 27 (47) |  |
| HADS Depression ^c^: |  |  |  |
| < 8 | 63 | 27 (43) | .42 |
| ≥ 8 | 24 | 8 (33) |  |
| HADS Anxiety ^c^: |  |  |  |
| < 8 | 41 | 17 (41) | .83 |
| ≥ 8 | 46 | 18 (39) |  |
| CAGE ^c^: |  |  |  |
| < 2 | 61 | 25 (41) | .83 |
| ≥ 2 | 26 | 10 (38) |  |
| **Variable** | **Vl > 50 (n = 52):**  **median (q1, q3)** | **Vl ≤ 50 (n = 35):**  **median (q1, q3)** | **P-value^b^** |
| Age | 37 (32, 44) | 32 (28, 39) | .04 |
| Stigma (out of 9)^c^ | 1 (0, 2) | 1 (0, 1) | .51 |
| Disclosure Worries (out of 100)^d^ | 60 (48, 62) | 60 (48, 80) | .94 |
| HAT-QoL Total (out of 100)^d^ | 62 (53, 71) | 68 (59, 75) | .11 |

^a^ Chi-squared test between the outcome and the variables listed in each row.

^b^ Mann–Whitney U (rank-sum) test between the outcome and the variables listed in each row.

^c^ Higher scores indicate more symptoms

^d^Lower scores indicate lower quality of life
